# Diagnostic efficiency of Xpert MTB/RIF assay for osteoarticular tuberculosis in patients with inflammatory arthritis in China

**DOI:** 10.1371/journal.pone.0198600

**Published:** 2018-06-01

**Authors:** Yuan Li, Wenyun Jia, Guohua Lei, Dan Zhao, Guirong Wang, Shibing Qin

**Affiliations:** 1 Department of Orthopedics, Beijing Tuberculosis and Thoracic Tumor Institute, Beijing, China; 2 Department of General Medicine, Beijing Tuberculosis and Thoracic Tumor Institute, Beijing, China; 3 Department of Pathology, Beijing Tuberculosis and Thoracic Tumor Institute, Beijing, China; 4 National Clinical Laboratory on Tuberculosis, Beijing Tuberculosis and Thoracic Tumor Institute, Beijing, China; Indian Institute of Technology Delhi, INDIA

## Abstract

**Background:**

Both osteoarticular tuberculosis (OA-TB) and inflammatory arthritis can lead to osteoarticular structural damage. These conditions exhibit similar symptoms, physical signs, and imaging features. Rapidly and accurately diagnosing OA-TB in patients with inflammatory arthritis presents a challenge to clinicians. Xpert MTB/RIF (Xpert) has been endorsed by the World Health Organization (WHO) as a rapid diagnostic tool for diagnosis of pulmonary and extrapulmonary TB. This study was designed to investigate diagnostic efficiency of Xpert for OA-TB in patients with inflammatory arthritis in China.

**Methods:**

A total of 83 consecutive patients with inflammatory arthritis and suspected OA-TB were enrolled prospectively from June 2014 to May 2018. Demographic, clinical, and biological data were recorded. Xpert assay, smear microscopy examination (smear), BACTEC MGIT 960 (MGIT 960), pathological examination, and T-SPOT.TB test were performed for each patient who received operations. Diagnostic efficiency of Xpert was evaluated based on a composite reference standard (CRS).

**Results:**

A total of 49 out of 83 patients with inflammatory arthritis and suspected OA-TB received operations, and 49 specimens were obtained during operations. According to CRS, 36 out of 49 patients with inflammatory arthritis were diagnosed with OA-TB, and 13 were not affected by the condition. Sensitivity of Xpert assay, smear, MGIT 960, pathological examination, and T-SPOT.TB test reached 66.70% (24/36), 25.00% (9/36), 30.55% (11/36), 47.22% (17/36), and 80.55% (29/36), respectively. Specificity of Xpert assay, smear, MGIT 960, and pathological examination was all 100% (13/13). Specificity of T-SPOT.TB test was 53.84% (7/13). Sensitivity of Xpert was higher than that of smear, MGIT 960 and pathological examination, but the sensitivity of Xpert was lower than that of T-SPOT.TB. Sensitivity of Xpert was statistically different from that of smear and MGIT 960 (*P*<0.001, *P* = 0.002), but the sensitivity of Xpert was not significantly different from that of pathological examination and T-SPOT.TB (*P* = 0.096, *P* = 0.181). Specificity of T-SPOT.TB was less than that of Xpert, smear, MGIT 960, and pathological examination, and the difference between them was statistically significant (*P* = 0.015). Among the 27 OA-TB patients with smear negative results, Xpert had the highest sensitivity, but sensitivity of Xpert was not significantly different from that of pathological examination and T-SPOT.TB (*P* = 0.413, *P* = 0.783). 2 of 36 OA-TB patients exhibited RIF resistance. Xpert was concordant with MGIT 960-based drug susceptibility testing (DST) in detecting rifampin (RIF) resistance.

**Conclusions:**

Xpert is an efficient tool with high sensitivity and specificity for OA-TB diagnosis in patients with inflammatory arthritis in high-TB prevalence countries. Compared with conventional methods, Xpert has two advantages: one is fast, and the other is able to provide RIF resistance information simultaneously.

## Introduction

TB has become a leading cause of death worldwide for decades. China is one of the 22 countries with the highest TB burden, and it hosts the second largest population of TB patients [1]. OA-TB accounts for 1%–3% of all TB cases. OA-TB damages osteoarticular structure, resulting in disability among patients. Control of OA-TB is dependent on accurate identification of cases and prompt treatment, thereby preserving more physical functions.

Inflammatory arthritis comprises a group of immune-mediated inflammatory diseases, including rheumatoid arthritis (RA), ankylosing spondylitis (AS), systemic lupus erythematosus arthritis (SLE-A), and psoriatic arthritis (PA). Autoimmune disorder and immunosuppressant application make patients susceptible to TB development [2]. OA-TB and inflammatory arthritis exhibit similar symptoms, physical signs, and imaging features. However, their treatments are contradictory. Therefore, rapid and accurate diagnosis of OA-TB in patients with inflammatory arthritis is vital.

Conventional diagnostic methods of OA-TB are less efficient; for example, acid-fast bacilli (AFB) smear microscopy shows low sensitivity; Mycobacterium TB culture is a time-consuming method; pathological examination needs tissue specimens; T-SPOT.TB displays low specificity in countries with high TB prevalence [3,4]. These conventional diagnostic methods cannot offer rapid results for DST. Xpert is an automated real-time polymerase chain reaction system that simultaneously detects TB and resistance to RIF in 2 h. Xpert performs excellently; hence, the WHO endorsed it for pulmonary TB diagnosis in 2010 and extrapulmonary TB diagnosis in 2013 [5,6].

To date, specialized studies rarely report usefulness of Xpert in OA-TB. Performance of Xpert for OA-TB diagnosis in patients with inflammatory arthritis does not have been reported. The present study was designed to evaluate diagnostic efficiency of Xpert assay for OA-TB in patients with inflammatory arthritis.

## Materials and methods

### Ethics statement

This study was established, according to the ethical guidelines of the Helsinki Declaration and was approved by the Human Ethics Committee of Beijing Tuberculosis and Thoracic Tumor Institute. The approval number was 2014–03. Written informed consent was obtained from individual participants.

### Patient categories

Consecutive patients with inflammatory arthritis and suspected OA-TB were enrolled prospectively in the Orthopedics Department of Beijing Chest Hospital (China) from June 2014 to May 2018. Pus and tissue specimens were obtained during operations. Blood specimens were obtained when all patients were admitted to the hospital.

Based on CRS, patients were categorized into two groups: (1) confirmed OA-TB cases (including A: mycobacterial culture positive and B: pathological result was tuberculosis and the patient responded well to anti-TB therapy), (2) non-TB cases (negative results for mycobacterial culture and all other tests for TB, and patient improved without receiving anti-TB treatment).

In this study, categories of inflammatory arthritis included RA, AS, SLE-A, and PA. Diagnosis of inflammatory arthritis (RA, AS, SLE-A, and PA) was based on the American College of Rheumatology criteria. Treatment drugs for inflammatory arthritis included immunosuppressants (methotrexate and leflunomide), corticosteroids, and nonsteroidal anti-inflammatory drugs. All patients preoperatively received anti-TB treatment for 4 weeks with isoniazid, RIF, ethambutol, and pyrazinamide.

### Smear and MGIT 960 culture

Smear: Pus specimens were processed directly and stained with auramine. Smears were examined by using light-emitting diode microscopy with the use of an eyepiece with10× magnifications and an objective with 40× magnification (total magnification, 400×). Each Smear was read by two laboratory technologists blindly and discordant results were confirmed by the 3rd laboratory technologist. The result was graded as negative, scanty and 1+, 2+ or 3+ depending on the number of bacilli under the microscope and reported according to WHO/ IUATLD guidelines[7].

MGIT 960 culture: MGIT 960 cultures were performed following manufacturer’s instructions. Briefly, pus specimens were decontaminated and diluted by treating with an equal volume of 2% sodium hydroxide and 0.5% N-acetyl-l-cysteine-sodium hydroxide for 15 min. After dilution, the tube was adjusted with 50 ml of phosphate buffer (0.1 M, pH 6.5) and centrifuged at 4000 × g for 20 min. Pellets were resuspended in 20 ml of phosphate buffer and recentrifuged. The final pellet was resuspended in 1 ml of phosphate buffer to provide sufficient volume for liquid culture in the MGIT 960 system (Becton Dickinson) for a maximum of 42 days, and monitored continuously. The MGIT 960 outcomes were reported as manufacturer’s instructions. Positive growth on MGIT tubes was examined microscopically for AFB. Standard DST with RIF was performed for positive cultures using the MGIT 960 IR kit (Becton Dickinson) following manufacturer’s instructions.

### Xpert assay

Pus specimens were tested by using Xpert according to manufacturer’s instructions (Cepheid, Sunnyvale, CA, USA). Briefly, 1 ml of pus specimen was mixed with 2 ml of Xpert sample reagent, vortexed for at least 10 s, and incubated at room temperature for 10 min. The mixture was vortexed again for another 10 s and incubated at room temperature for 5 min. A total of 2 ml of the mixture was transferred to the Xpert cartridge and loaded into the GeneXpert instrument. Automatic detection procedure was carried out. The result was reported automatically.

### Pathological examination

Tissue specimens collected during operations were fixed in neutral formalin, dehydrated, and subsequently paraffin-embedded. Paraffin-embedded tissues were sliced. Afterward, 4 μm sections were stained with hematoxylin and eosin solution and observed using light microscopy for pathomorphological changes. AFB test was performed only when the pathologists needed further information. Briefly, 4.0 μm sections were dewaxed by dimethylbenzene, then sequentially washed with 95%, 90%, 85%, 70% ethanol, and finally by de-ion water. After drying, the slides were stained by standard Zeihl-Neelsen method, and AFB was detected under an oil immersion lens (×1000). Each pathological section was read by two experienced pathologists blindly and the discordant consequence was confirmed by the 3rd experienced pathologist. The result was reported according to WHO/ IUATLD guidelines[7].

The pathological diagnostic criteria (1) confirmed TB: AFB was observed in the lesion, typical chronic granulomatous inflammation, with or without caseous necrosis, were also observed, (2) no TB: neither granulomatous inflammation nor caseous necrosis were observed, and AFB was not observed in the lesion.

### T-SPOT.TB test

Blood T-SPOT.TB test (Oxford Immunotec Ltd., Abingdon, UK) was performed according to manufacturer’s instructions. Briefly, peripheral blood mononuclear cells (PBMCs) were separated from peripheral venous blood of each patient. PBMCs were isolated and incubated with two antigens (early secretory antigenic target 6 and culture filtrate protein 10). The procedure was performed in plates precoated with anti-interferon-γ antibodies at 37°C for 18 h. After application of alkaline phosphatase-conjugated secondary antibody and chromogenic substrate, the number of spot-forming cells in each well was automatically counted with a CTL ELISPOT system (CTL-ImmunoSpot® S5 Versa Analyzer). The response of stimulated cultures was considered positive when one or both test wells contained at least six more spots than the negative control wells or had at least twice as many as spot-forming cells as the negative control wells. Criteria for positive, negative, and indeterminate outcomes were recommended by the manufacturer.

### Date management and statistical analysis

All data were entered into a Microsoft Office Excel file. Sensitivity, specificity, positive predictive value (PPV) and negative predictive value (NPV) were computed to evaluate diagnostic performance for different OA-TB test methods. The statistical difference between different OA-TB test methods was compared using Pearson χ^2^ tests or Fisher’s exact tests, as appropriate. All tests of significance were two-tailed, and *p* < 0.05 was considered statistically significant. Analysis was performed using the commercial statistical software SPSS version 13.0 (SPSS Inc, Chicago, IL, USA).

## Results

### Patient characteristics

A total of 83 consecutive patients with inflammatory arthritis and suspected OA-TB were enrolled prospectively. Subsequently, 49 of the 83 patients received operations, and 49 pus and tissue specimens were obtained during operations. Ultimately, 49 patients were included in this study. According to CRS, 36 patients were diagnosed with OA-TB, whereas 13 patients showed no evidence of TB. Table 1 shows demographic characteristics of patients in different categories. Among the 49 patients, 19, 10, 11, and 9 patients exhibited RA, AS, SLE-A, and PA, respectively.

**Table 1:** Clinical characteristics of 49 studied patients.

Clinical characteristics	OA-TB (n = 36)	Non-OA-TB (n = 13)
Age, years (mean ± SD)	53.28±15.97	53.77±15.97
Female, n (%)	23 (63.88)	7 (53.84)
Spine lesion, n (%)	15 (41.67)	5 (38.46)
Joint lesion, n (%)	21 (58.33)	8 (61.54)
Disease diagnosis, n (%)		
Rheumatoid arthritis	14 (38.89)	5 (38.46)
Ankylosing spondylitis	7 (19.44)	3 (23.08)
Systemic lupus erythematosus arthritis	9 (25.00)	2 (15.38)
Psoriatic arthritis	6 (16.67)	3 (23.08)
Disease duration		
Inflammatory arthritis, years (range)	11.24 ±9.11(0.2–40)	14.59±12.08 (0.2–43)
OA-TB, months (range)	13.18±12.82 (2–48)	0
Immunosuppressive therapy, n (%)	27 (75.00)	9(69.23)
Previous biologics, n (%)	0 (0)	0 (0)
Corticosteroids, n (%)	24 (66.67)	7 (53.84)
NSAIDs, n (%)	19 (52.78)	7(53.84)
Duration of antiTB treatment prior to sample collection, weeks	4	0

N: number of patients; OA-TB: Osteoarticular tuberculosis; NSAIDs: nonsteroidal anti-inflammatory drugs

### Sensitivity and specificity of Xpert assay, smear, MGIT 960, pathological examination, and T-SPOT.TB

According to CRS, sensitivity of Xpert assay, smear, MGIT 960, pathological examination, and T-SPOT.TB test totaled 66.70% (24/36), 25.00% (9/36), 30.55% (11/36), 47.22% (17/36), and 80.55% (29/36), respectively. Specificity of Xpert assay, smear, MGIT 960, and pathological examination was all 100% (13/13). Specificity of T-SPOT.TB test was 53.84% (7/13). Table 2 shows performances of Xpert MTB/RIF assay and conventional diagnostic methods for OA-TB diagnosis in patients with inflammatory arthritis.

**Table 2:** Performance of Xpert MTB/RIF assay and conventional diagnostic methods for OA-TB diagnosis in patients with inflammatory arthritis.

Methods	Sensitivity (%, N, 95% CI)	Specificity (%, N, 95% CI)	PPV (%, N, 95% CI)	NPV (%, N, 95% CI)
Xpert	66.70% (24/36) (49–81)	100% (13/13) (75–100.0)	100% (24/24) (86.0–100.0)	52.00% (13/25) (31–72)
Smear	25.00% (9/36) (12–42.0)	100% (13/13) (75–100.0)	100% (9/9) (66.0–100.0)	32.50% (13/40) (19–49)
MIGT 960	30.55% (11/36) (16–48)	100% (13/13) (75–100.0)	100% (11/11) (72.0–100.0)	34.21% (13/38) (20–51)
Pathological	47.22% (17/36) (30–65)	100% (13/13) (63.0–100.0)	100% (17/17) (80.0–100.0)	40.62% (13/32) (24–59)
T-SPOT.TB	80.55% (29/36) (64.0–92.0)	53.84% (7/13). (25.0–81.0)	82.85% (29/35) (66.0–100.0)	50.00% (7/14) (23–77)

N: number of patients, PPV: positive predictive value, NPV: Negative predictive value, CI: confidence interval; OA-TB: Osteoarticular tuberculosis

Sensitivity of Xpert was higher than that of smear, MGIT 960 and pathological examination, but the sensitivity of Xpert was lower than that of T-SPOT.TB. Sensitivity of Xpert was statistically different from that of smear and MGIT 960 (*P*<0.001, *P* = 0.002), but the sensitivity of Xpert was not significantly different from that of pathological examination and T-SPOT.TB (*P* = 0.096, *P* = 0.181). T-SPOT.TB was more sensitive than pathological examination, and the difference between them was statistically significant (*P* = 0.003). The specificity of the T-SPOT.TB was less than that of Xpert, smear, MGIT 960, and pathological examination, and the difference between them was statistically significant (*P* = 0.015). Table 3 shows statistical analysis results between different detection methods.

**Table 3:** Statistical results of sensitivity and specificity between different detection methods.

	T-SPOT.TB	Smear	MIGT 960	Pathological	Xpert
Xpert	χ^2^ = 1.787 P = 0.181	χ^2^ = 12.587 P<0.001	χ^2^ = 9.396 P = 0.002	χ^2^ = 2.776 P = 0.096	
Pathological	χ^2^ = 8.669 P = 0.003				
T-SPOT.TB		P = 0.015^a^	P = 0.015^a^	P = 0.015^a^	P = 0.015^a^

^a^ the P value is calculated by Fisher exact test

In this study, 27 of 36 patients with OA-TB had negative smear results. Among the 27 patients with smear negative, the sensitivity of Xpert assay, MGIT 960, pathological examination, and T-SPOT.TB was 59.26% (16/27), 14.81% (4/27), 48.15% (13/27), 55.55% (15/27) respectively. Xpert had the highest sensitivity, but sensitivity of Xpert was not significantly different from that of pathological examination and T-SPOT.TB (*P* = 0.413, *P* = 0.783). Sensitivity of Xpert was higher than that of MGIT 960, which was statistically significant (*P* = 0.001). Table 4 shows statistical analysis results between different detection methods of 27 smear negative patients.

**Table 4:** Statistical results of sensitivity between different detection methods of 27 smear negative patients.

	MIGT 960	Pathological	T-SPOT.TB
Xpert	χ^2^ = 11.435 P = 0.001	χ^2^ = 0.670 P = 0.413	χ^2^ = 0.076 P = 0.783

### Detection of RIF resistance

Xpert detected 2 RIF-resistant patients among 36 OA-TB patients. Standard MGIT 960 DST was performed for the 6 mycobacterial TB isolates were recovered from positive culture pus samples. One RIF-resistant isolate was obtained from the 11 mycobacterial TB isolates were identified by MGIT 960 DST. Xpert was concordant with MGIT 960 DST with regard to detection of RIF resistance.

## Discussion

In high TB prevalence countries, such as China, patients with inflammatory arthritis show susceptibility to TB development because of autoimmune disorders and immunosuppressant application. Inflammatory arthritis combined with OA-TB is rare, but it is often misdiagnosed. So we should pay greater attention to it. To date, imaging examinations (such as computed tomography and magnetic resonance imaging) and immunological tests (such as tuberculin skin test and T-SPOT.TB) are the most common diagnostic technologies for OA-TB in China [8–9]. The use of these diagnostic technologies in detecting OA-TB in patients with inflammatory arthritis can be confusing, because of similar imaging features between these two diseases and high latent TB infection rate in China. Hence, rapid and accurate diagnosis of OA-TB in patients with inflammatory arthritis is a challenge for physicians. Build on clinical practice, this study selected the commonly used diagnostic techniques of OA-TB, to evaluate diagnostic efficiency of Xpert for OA-TB in patients with inflammatory arthritis.

Sufficient validations were performed to evaluate the use of Xpert in diagnosis of pulmonary TB and extrapulmonary TB [6]. Nevertheless, no evidence demonstrates its usefulness in inflammatory arthritis patients with OA-TB. The present study showed that sensitivity and specificity of Xpert in diagnosing OA-TB patients with inflammatory arthritis reached 66.70% and 100%, respectively. Sensitivity of Xpert was higher than that of smear, MGIT 960 and pathological examination, but the sensitivity of Xpert was lower than that of T-SPOT.TB. Sensitivity of Xpert was statistically different from that of smear and MGIT 960 (*P*<0.001, *P* = 0.002), the sensitivity of Xpert was not significantly different from that of pathological examination and T-SPOT.TB (*P* = 0.096, *P* = 0.181). In general, the sensitivity of Xpert in this study was right on the same level as that of pathological examination and T-SPOT.TB, but superior to smear and MGIT 960. In this study, Xpert, smear, MGIT 960 and pathological examination all had excellent specificity and were at the same level, and better than T-SPOT.TB. Previous studies had demonstrated that the sensitivity of Xpert was 71.2% -82%, and the specificity of Xpert was 100% [10–12]. Sensitivity and specificity of Xpert in this study were consistent with previously reported results in OA-TB patients. Autoimmune disorders and immunosuppressant application appeared to exert no effect on sensitivity and specificity of Xpert.

Among the 27 OA-TB patients with smear-negative results, Xpert had the highest sensitivity, but sensitivity of Xpert was not significantly different from that of pathological examination and T-SPOT.TB (*P* = 0.413, *P* = 0.783). The sensitivity of Xpert, pathological examination and T-SPOT.TB was right on the same level in smear-negative patients, but superior to MGIT 960. Previous study had reported that the sensitivity of Xpert was 47.7% for smear-negative extrapulmonary specimens[13].

In this study, the sensitivity of smear, MGIT 960 was low. The reasons for the low sensitivity of the smear may be due to the paucibacillary nature of OA-TB specimens, and smear detection technology itself needs high concentration of mycobacterium TB[14]. MGIT 960 culture needs live mycobacterium TB. The reasons for the low sensitivity of MGIT 960 are probably due to anti-TB treatment for 4 weeks before specimen collection, which may has killed the majority of live mycobacterium TB, in addition to the paucibacillary nature of OA-TB specimens. The reason for the high sensitivity of Xpert may be that Xpert detects the genes of mycobacterium TB, not mycobacterium TB. Even though the mycobacterium TB has died, the gene is still there. In this study, specificity of T-SPOT.TB was only 53.84%. Low specificity of T-SPOT.TB may be caused by high TB prevalence in China, where numerous individuals are infected with latent TB. Thereby in high TB prevalence countries, low specificity of T-SPOT.TB reduces its diagnostic efficiency for OA-TB in patients with inflammatory arthritis. In this study, the sensitivity and specificity of the pathological examination were at the same level as Xpert. Nonetheless, pathological examinations consume several days before obtaining results and cannot provide DST result[15]. These factors may lead to delaying treatment of OA-TB patients with inflammatory arthritis. Xpert can detect RIF-resistant gene mutations, thereby providing information whether patients exhibit RIF resistance. Xpert consumes only 2 h to obtain results, but conventional DST methods (Lowenstein-Jensen and MIGT 960 cultures) require much considerable time. Delayed discovery of RIF resistance in patients with OA-TB may lead to disease deterioration.

This study showed that Xpert had both high sensitivity and specificity for OA-TB diagnosis in patients with inflammatory arthritis in high-TB prevalence countries. Compared with conventional methods, the advantages of Xpert are time-saving and simultaneous detection of RIF resistance. These two advantages are very practical in clinical practice. Thus, Xpert can rapidly and accurately diagnose OA-TB in patients with inflammatory arthritis, with information that whether mycobacterium TB exists RIF resistance. Treatments of OA-TB and inflammatory arthritis are contradictory, so a clear diagnosis of OA-TB in patients with inflammatory arthritis is important. Build on the results of Xpert, clinicians can distinguish between patients with inflammatory arthritis combined with OA-TB or inflammatory arthritis only. Clinicians can adjust the amount of immunosuppressant application and add an effective anti-TB treatment to preserve many physical functions.

In summary, Xpert is a rapid and effective diagnostic tool for OA-TB in patients with inflammatory arthritis. This technology shows high sensitivity and specificity. Compared with conventional methods, Xpert has two advantages: one is fast, and the other is able to provide RIF resistance information simultaneously. But the sample size of this study is small. In the future, we will need a larger sample size study.

## References

[1] World Health Organization. Global Tuberculosis Report 2013. Geneva, Switzerland. WHO/HTM/. TB/2013.11.

[2] Tikly M, Navarra S. Lupus in the developing world–Is it any different? Best Pract Res Clin Rheumatol 2008;22:643–55. doi: 10.1016/j.berh.2008.05.003 18783742 10.1016/j.berh.2008.05.003

[3] Chegou NN, Hoek KG, Kriel M, Warren RM, Victor TC, Walzl G. Tuberculosis assays: past, present and future. Expert Rev Anti Infect Ther, 2011; 9:457–69. doi: 10.1586/eri.11.23 21504402 10.1586/eri.11.23

[4] Chen CH, Chen YM, Lee CW, Chang YJ, Cheng CY, Hung JK. Early diagnosis of spinal tuberculosis. J Formos Med Assoc.2016; 115: 825–836. doi: 10.1016/j.jfma.2016.07.001 27522334 10.1016/j.jfma.2016.07.001

[5] World Health Organization Roadmap for rolling out Xpert MTB/RIF for rapid diagnosis of TB and MDR-TB;2010 Geneva, Switzerland:WHO, 2013. Availabe:online: www.who.int/tb/laboratory/roadmap_xpert_mtb-rif.pdf

[6] World Health Organization. Automated real-time nucleic acid amplification technology for rapid and simultaneous detection of tuberculosis and rifampicin resistance: Xpert MTB/RIF assay for the diagnosis of pulmonary and extrapulmonary TB in adults and children. Geneva: World Health Organization 2013. 25473701

[7] Mohammed A, Gisela B, Fadila B, David D, Lanfranco F, Knut F, et al Technical guide: sputum examination for tuberculosis by direct microscopy in low income countries, 5th ed, Paris, France: International Union against Tuberculosis and Lung Disease; 2000.

[8] Chen ST, Zhao LP, Dong WJ, Gu YT, Li YX, Dong LL, et al The Clinical Features and Bacteriological Characterizations of Bone and Joint Tuberculosis in China. Sci Rep.2015;5:11084 doi: 10.1038/srep11084 26053666 10.1038/srep11084 PMC4459174

[9] World Health Organization. Use of Tuberculosis Interferon-Gamma Release Assays (IGRAs) in Low- and Middle-Income Countries: Policy Statement. Geneva: World Health Organization; 2011. 26269875

[10] Gu Y, Wang G, Dong W, Li Y, Ma Y, Shang Y, et al Xpert MTB/RIF and GenoType MTBDRplus assay for the rapid diagnosis of bone and joint tuberculosis.Int J Intfect Dis.2015,36:27–30 10.1016/j.ijid.2015.05.014 26004172

[11] Held M, Laubscher M, Mears S, Dix-Peek S, Workman L, Zar H, et al Diagnostic Accuracy of the Xpert MTB/RIF Assay for Extrapulmonary Tuberculosis in Children Musculoskeletal Infections. Pediatr Infect Dis J.2016;35:1165–1168. doi: 10.1097/INF.0000000000001271 27286562 10.1097/INF.0000000000001271 PMC5071124

[12] Arockiaraj J, Michael JS, Amritanand R, David KS, Krishnan V. The role of Xpert MTB/RIF assay in the diagnosis of tuberculosis spondylodiscitis. Eur Spine J.2017; doi: 10.1007/s00586-017-5076-9 28391384 10.1007/s00586-017-5076-9

[13] Zeka AN, Tasbakan S, Cavusoglu C. Evaluation of the GeneXpert MTB/RIF assay for rapid diagnosis of tuberculosis and detection of rifampin resistance in pulmonary and extrapulmonary specimens. J Clin Microbiol. 2011;49(12):4138–41. doi: 10.1128/JCM.05434-11 21956978 10.1128/JCM.05434-11 PMC3232962

[14] Theron G, Peter J, Calligaro G, Meldau R, Hanrahan C, Khalfey H, et al Determinants of PCR performance(Xpert MTB/RIF),including bacterial load andinhibition,for TB diagnosis using specimens from different body compartments.Sci Rep.2014;4:5658 doi: 10.1038/srep05658 25014250 10.1038/srep05658 PMC5375978

[15] Denis-Delpierre N, Merrien D, Billaud E, Besnier JM, Duhamel E, Hutin P, et al [Extrapulmonary tuberculosis in the central western region. Retrospective study of 217 cases (Gericco1991–1993)]. Presse Med. 27, 341–346 (1998). 9767996

